# Comparison of inpatient psychiatric care for SARS-CoV-2 positive and negative adults in Vienna

**DOI:** 10.1192/j.eurpsy.2024.763

**Published:** 2024-08-27

**Authors:** A. Erfurth

**Affiliations:** 1st Department of Psychiatry and Psychotherapeutic Medicine, Klinik Hietzing, Vienna, Austria

## Abstract

**Introduction:**

The structure of psychiatric care has undergone many changes in recent decades. In addition, the SARS-CoV-2 pandemic has posed specific challenges for inpatient psychiatric care. In Vienna, the admission of SARS-CoV-2 positive psychiatric patients has been centralised in one department, the 1st Department of Psychiatry and Psychotherapeutic Medicine, Klinik Hietzing.

**Objectives:**

It will be investigated to what extent the admissions of SARS-CoV-2 positive and negative patients differ with regard to age, gender, diagnosis, need for involuntary admission, medication, duration of treatment, country of birth and the question of where the patients come from and where they are discharged to.

**Methods:**

Between 15 March 2020 and 21 May 2022 (start and end of cohorting of all Vienna SARS-CoV-2 positive inpatient psychiatric patients in one department), 338 SARS-CoV-2 positive and 1312 SARS-CoV-2 negative patients were treated as inpatients at the 1st Department of Psychiatry and Psychotherapeutic Medicine of the Klinik Hietzing.

**Results:**

The results of the study will be shown.

**Conclusions:**

The SARS-CoV-2 pandemic has presented an outstanding challenge to inpatient psychiatry. An accurate portrayal of differences in the treatment of positive and negative patients is of importance for assessing the impact of the pandemic.

**Disclosure of Interest:**

None Declared

